# *Staphylococcus aureus* CC398 Lineage of the Human Clade Isolated from Bloodstream Infection and Colonization and Spread among Brazilian Patients Hospitalized during the COVID-19 Pandemic

**DOI:** 10.1007/s00284-026-04996-x

**Published:** 2026-06-30

**Authors:** Thaís Campos Macharete, Tamara Lopes Rocha de Oliveira, Gabriel Freire Igari, Bruna Marques de Souza, Simone Aranha Nouér, Fernanda Sampaio Cavalcante, Kátia Regina Netto dos Santos

**Affiliations:** 1 Departamento de Microbiologia Médica, Instituto de Microbiologia Paulo de Góes, Universidade Federal do Rio de Janeiro, Rio de Janeiro, Brazil; 2 Departamento de Doenças Infecciosas e Parasitárias, Hospital Universitário Clementino Fraga Filho, Universidade Federal do Rio de Janeiro, Rio de Janeiro, Brazil; 3 Departamento de Clínica Médica, Instituto de Ciências Médicas, Centro Multidisciplinar de Macaé, Universidade Federal do Rio de Janeiro, Macaé, Brazil; 4 Laboratório de Infecção Hospitalar, Departamento de Microbiologia Médica, Instituto de Microbiologia Paulo de Góes, Universidade Federal do Rio de Janeiro, CCS, Bloco I, Sala I2‑010 ‑ Av. Carlos Chagas Filho 373, Cidade Universitária, Rio de Janeiro, CEP 21941‑590 RJ Brazil

## Abstract

Community lineages of *Staphylococcus aureus* emerging in hospitals, such as CC398, have raised concerns. *S. aureus* CC398 isolates from bloodstream infections (BSI) and nasal colonization of hospitalized patients, during the COVID-19 pandemic, between March/2020 and September/2021, were characterized for antimicrobial susceptibility by disk diffusion test. Spa types and Sequence Type (ST) were analyzed by PCR/sequencing, and genes of resistance, virulence, and human immune evasion cluster (IEC) were accessed by PCR. Of a total of 118 CC398 isolates, 104 were from nasal swabs (4 MRSA and 100 MSSA), and 14 MSSA were from BSI. Forty (33.9%) patients presented COVID-19, and prior antimicrobial use was common among those colonized (*p* < 0.05). High resistance rates were found for penicillin (PEN, 92.4%), erythromycin (ERY, 95.8%), clindamycin (CLI, 90.7%) and gentamicin (GEN, 51.7%), and the iMLS_B_ phenotype was commonly detected. All four nasal MRSA and 47.4% of MSSA isolates were multidrug-resistant. The CLI/ERY/GEN/PEN/*erm*T/*erm*C and CLI/ERY/*erm*T resistance profiles were frequent in nasal (45.2%) and BSI (57.1%) isolates, respectively. The *erm*C gene was more found in nasal (93.3%) than in BSI (7.1%) isolates (*p* < 0.05). Most of 95% of the isolates presented the *fnbA*, *fnbB*, *cna*, and *ermT* genes. The *spa* type t1451 was detected in 91.5% of isolates, and the human clade was associated with 98.3%, with IEC type C being prevalent. MSSA-CC398 isolates of the human clade were predominant among hospitalized patients during the pandemic. The increased selective pressure exerted on these isolates, previously resistant to macrolides, may have favored their dissemination in the environment.

**Supplementary Information:**

The online version contains supplementary material available at 10.1007/s00284-026-04996-x.

## Introduction

*Staphylococcus aureus* is a leading cause of mortality worldwide and one of the most frequent pathogens isolated in bloodstream infections (BSI), and nasal colonization has been associated with an increased risk of developing these infections [1]. The persistence and spread of *S. aureus* in healthcare settings is due to a lack of hand hygiene compliance, and the bacterium’s remarkable ability to resist selective pressure imposed by antimicrobials and disinfectants [2].

In recent decades, many community-associated clonal complexes (CC) of *S. aureus* have successfully adapted to hospital environments in diverse geographic regions, such as CC5and CC8 [3]. Lastly, CC398 has also emerged as a lineage of increasing concern in some countries [4–5]. Isolates belonging to CC398 were first identified in the Netherlands by Voss et al. [6] and were methicillin-resistant *S. aureus* (MRSA), recovered from pigs and individuals involved in pig farming. Since then, livestock have been reported as a reservoir for a MRSA lineage known as livestock-associated MRSA (LA-MRSA) clade, and the presence of the *tet*M gene and absence of the *scn* gene are classic characteristics of this clade [4, 6].

Since 2020, a new clade of the CC398 lineage have been increasingly reported as causing invasive infections in patients with no history of exposure to livestock, suggesting human-to-human transmission [5, 7]. These strains were characterized as methicillin-susceptible *S. aureus* (MSSA) and by the presence of the φSa3 prophage, which carries the immune evasion cluster (IEC) [4]. This cluster contains the *scn*,* sak*,* chp*, and *sea* (or *sep*) genes, which encode proteins capable of evading the immune system and play a fundamental role in human adaptation [8], and thus, these strains were named human-adapted (HU) clade. More recently, the global CC398 phylogeny presented by Chen et al. [9] suggested the presence of some human CC398 isolates in the animal source clade and vice versa, which further supports the spread of CC398 between animals and humans.

Despite the global relevance of CC398 and previous reports of its presence worldwide, including South America [10], there are still few reports about its isolation from human clinical sources [5, 9–12]. In a Spanish multicenter study conducted in 17 hospitals in 2020, with 1022 *S. aureus* isolates from bloodstream infections, 1% of the MRSA and 4.3% of the MSSA isolates belonged to the CC398 lineage [5]. Regarding colonization by CC398 *S. aureus* a study conducted in France from 2019 to 2020 detected 24.6% of individuals from community and 18.3% of hospitalized patients carrying this pathogen [13]. In Brazil, the CC398 lineage has already been described as cause of fatal pneumonia in a cancer patient [12]. In 2017, André-Neto et al. evaluated nasal swabs of 1852 children, both outpatients and inpatients, and only 0.3% of them were colonized by CC398 MRSA isolates [14].

During the COVID-19 pandemic, a significant increase in antimicrobial use combined with possible failures in infection control and prevention measures were observed, aspects that may have created selective pressure on microorganisms, as previously reported [15, 16]. However, reports of the isolation and characterization of CC398 isolates obtained during the pandemic are still rare. Furthermore, most countries do not perform molecular characterization of MSSA isolates, and therefore the effective role of this pathogen is largely unknown. We recently conducted studies on BSI [17] and nasal colonization [18] in patients at a hospital in Rio de Janeiro during the pandemic and found a large number MSSA isolates that were later identified as CC398. Due to the importance of invasive infections and colonization reservoirs involving *S. aureus* isolates the aim of this study was to characterize these CC398 isolates from BSI and nasal colonization regarding antimicrobial resistance, presence of resistance, virulence and human immune evasion cluster (IEC) genes and the genetic background.

## Materials and Methods

### Setting, CC398 Isolates, and Clinical and Sociodemographic Data Associated with the Patients

We conducted prospective cohort studies during the COVID-19 pandemic to evaluate *S. aureus* isolates from BSI [17] and nasal surveillance swabs of patients admitted to the Intensive Care Units [18], between March 2020 and September 2021, in adult individuals admitted to the Clementino Fraga Filho University Hospital, with about 300 active beds, in Rio de Janeiro, Brazil. Nasal sampling was performed up to 72 h after the patient’s admission. 

Among the *S. aureus* isolates obtained from this large study, 63 were from BSI and 255 were from nasal swabs. Among the BSI *S. aureus* isolates, 33 were MSSA isolates without characterization. Among nasal isolates 162 MSSA had also not been characterized, and four MRSA isolates were identified as CC398. Therefore, all MSSA isolates from BSI and nasal swabs were evaluated to detect their clonal complex (CC) by PCR-based restriction modification (RM) test [19, 20], and then all those belonging to CC398 were selected for further characterization in the present study.

All MRSA isolates had been confirmed for the presence of the *mec*A gene by PCR and analyzed by PFGE and multilocus sequence typing (MLST) methods [18].

The medical records of each colonized patient included in the study were accessed, and the following information was collected: age, gender, length of hospitalization, azithromycin use before admission, antimicrobial use before admission and during hospitalization and outcome. The research data was collected confidentially, ensuring the privacy of the subjects.

### Antimicrobial Susceptibility Tests

All *S. aureus* CC398 isolates were evaluated by their antimicrobial susceptibility using the disk-diffusion method, according to the CLSI guidelines [22]. The following disks were used: cefoxitin (30 µg), ciprofloxacin (5 µg), clindamycin (2 µg), erythromycin (15 µg), gentamycin (10 µg), linezolid (30 µg), mupirocin (200 µg), penicillin G (10U), rifampicin (5 µg), trimethoprim-sulfamethoxazole (25 µg) and tetracycline (30 µg) (Oxoid, Cambrigde, UK). *S. aureus* ATCC 25,923 was used as control for the test. The test for determining the induced resistance phenotype (iMLSb) was performed by proximity of the clindamycin disk to the erythromycin disk. (D test), according to CLSI guidelines [21]. Multidrug resistance (MDR) was defined as the presence of resistance to at least three classes of antimicrobials in addition to penicillin.

### Detection of Virulence and Resistance Genes

Bacterial DNA was extracted using Chelex-100 resin [22]. The presence of virulence genes, including those encoding adhesins and toxins, as well as macrolide-associated genes and *tet* genes, were detected by PCR for all CC398 *S. aureus* isolates. All primers, cycle programs and references are provided in the Supplementary Table 1 [8, 23–31].

### Genotype Tests

All MSSA isolates from BSI, and nasal swabs were evaluated to detect their clonal complex (CC) as indicated by PCR-based restriction modification (RM) test. All *S. aureus* presumptively belonging to CC398 (as indicated by RM test) were submitted to *spa* typing [32], while the confirmatory MLST method [33] was conducted for representative MSSA isolates.

### Immune Evasion Cluster (IEC) Type and Clades

The presence of the genes *scn*, *chp*, *sak*, *sea*, and/or *sep*, which comprise the immune evasion cluster (IEC) and are common in human strains, was investigated in all CC398 isolates by PCR (Supplementary Table 1). Based on the observed genetic patterns, isolates were classified into different IEC types, according to van Wamel and coworkers [8].

Discrimination between clades of the CC398 was performed based on the presence of genes associated with HU (*scn*) and LA (*tet*M) clades.

### Statistical Analysis

GraphPad Prism software, version 8 (GraphPad*, San Diego, CA, USA), was used to perform the statistical analysis. The variables were analyzed using Fisher’s exact, chi-square or Mann-Whitney U tests. Results with *p* < 0.05 were considered statistically significant.

## Results

### CC398 Isolates and Clinical and Sociodemographic Data Associated with the Patients

The clinical and sociodemographic data associated with the 118 patients who presented bloodstream infection (42%, 14/33 patients with MSSA isolates) or nasal colonization (62%, 100/162 patients with MSSA isolates) by *S. aureus* CC398 are described in Table 1, as well as four nasal MRSA isolates. Among them, 40 (33.9%) patients were diagnosed with COVID-19. Age and gender were similar for patients with and without COVID-19 in both situation, nasal colonization and BSI. Most BSI (71.4%) was classified as Primary and the average length of hospitalization for patients with COVID-19 (32.3 days) was shorter than for patients without the disease (68.8 days) (*p* = 0.4535).

**Table 1:** Description of the clinical and sociodemographic data associated with 118 patients that presented bloodstream infection or nasal colonization by CC398 *Staphylococcus aureus* during the COVID-19 pandemic

Patients Characteristics ^a^	Total	COVID-19 ICU	Non-COVID-19 ICU	*p*
Bloodstream infection	*n* = 14	*n* = 4	*n* = 10	
Age -years; Mean (SD)	66.3 (16.6)	68 (83.2)	68.2 (12.4)	0.6583
Sex -female; N (%)	7 (50)	2 (50)	5 (50)	1
Classification – Primary BSI; N (%)	10 (71.4)	3 (75)	7 (70)	1
Average hospitalization (days)	58.4	32.3	68.8	0.4535
Previous use of Azithromycin; N (%)	5 (35.7)	3 (75)	2 (20)	0.0949
Previous use of antimicrobials; N (%)	7 (50)	3 (75)	4 (40)	0.5594
Outcome death; N (%)	10 (71.4)	3 (75)	7 (70)	1
**Nasal colonization**	*n* = 104	*n* = 36	*n* = 68	
Age -years; Mean (SD)	56.5 (17)	56.3 (16.9)	56.4 (17)	0.4327
Sex -female; N (%)	52 (50)	17 (47.2)	35 (51.5)	0.6802
Average hospitalization (days)	50.1	50.9	50.1	0.1784
Previous use of Azithromycin; N (%)	11 (10.6)	8 (22.2)	3 (4.4)	0.0078
Previous use of antimicrobials; N (%)	59 (56.7)	29 (80.6)	30 (44.1)	0.0004
Outcome death; N (%)	40 (38.5)	23 (63.9)	17 (25)	0.0001

For colonized patients, the mean hospitalization time was similar for both types of patients, with and without COVID-19. However, the previous use of antimicrobials in general was significantly higher among those with COVID-19 (80.6%, 29/36) when compared to patients without the disease (44.1%, 30/68) (*p* = 0.0004), as well as for those who used Azithromycin, with values of 22.2% (8/36) and 4.4% (3/68), respectively (*p* = 0.0078).

### Antimicrobial Susceptibility and Resistance Genes

Antimicrobial susceptibility testing showed that CC398 isolates had high rates of resistance to penicillin G (92.4%), erythromycin (95.8%), clindamycin (90.7%), and gentamicin (51.7%) (Supplementary Table 2). The iMLS_B_ phenotype was detected in almost 90% of the isolates. Resistance to cefoxitin (4 isolates), ciprofloxacin (3) and tetracycline (3) were found only among nasal isolates. All isolates were susceptible to linezolid, mupirocin, rifampicin and trimethoprim-sulfamethoxazole. All four MRSA isolates presented MDR profiles, as well as 47.4% of MSSA isolates. No significant difference was identified between isolates from patients with and without COVID-19.

Fifteen profiles of antimicrobial phenotypic/genotypic resistance were identified among nasal isolates (Table 2). The most frequent profile was CLI/ERY/GEN/PEN/*erm*T/*erm*C (45.2%), followed by CLI/ERY/PEN/*erm*T/*erm*C (31.7%). Among BSI isolates, the CLI/ERY/*erm*T profile was the most found (57.1%).

**Table 2:** Correlation between phenotypic and genotypic characteristics of antimicrobial resistance in 118 CC398 *Staphylococcus aureus* isolates from bloodstream infection and nasal swabs

Phenotypic profile^a^	Genotypic Profile	*N* (%) of isolates
BSI		*n* = 14
MSSA		
CLI/ERY	*erm*T	8 (57.1)
CLI/ERY	*erm*T, *erm*C	1 (7.1)
CLI/ERY/GEN	*erm*T	3 (21.4)
ERY/GEN	*erm*T	1 (7.1)
GEN	-	1 (7.1)
**Nasal swab**		*n* = 104
**MRSA**		
CEF/CLI/ERY/PEN	*erm*T	1 (1)
CEF/CLI/ERY/PEN	*erm*T/*erm*C	1 (1)
CEF/CIP/CLI/ERY/PEN/TET	*erm*T/*erm*C/*tet*K	1 (1)
CEF/CIP/CLI/ERY/GEN/PEN/TET	*erm*T/*erm*C/*tet*M	1 (1)
**MSSA**		
CLI/ERY/GEN/PEN	*erm*T, *erm*C	47 (45.2)
CLI/ERY/GEN/PEN	*erm*T	2 (1.9)
CLI/ERY/PEN	*erm*T, *erm*C	33 (31.7)
CLI/ERY	*erm*T, *erm*C	7 (6.7)
ERY/GEN/PEN	*erm*T, *erm*C	3 (2.9)
PEN	-	3 (2.9)
CIP/CLI/GEN/PEN	*erm*T, *erm*C	1 (1)
CLI/ERY/GEN	*erm*T, *erm*C	1 (1)
ERY/PEN	*erm*T, *erm*C	1 (1)
GEN/PEN	*erm*T	1 (1)
ERY	*erm*T, *erm*C	1 (1)

^a^ isolates classified as resistant and intermediate resistant, according to CLSI, 2020; BSI: bloodstream infection. CEF: Cefoxitin; CIP: Ciprofloxacin; CLI: Clindamycin; ERY: Erythromycin; GEN: Gentamicin; PEN: Penicillin G; TET: Tetracycline

The resistance genes *erm*T and *erm*C were detected in 114 (96.6%) and 98 (83.1%) CC398 isolates, respectively. The *erm*C gene was more found in nasal isolates (93.3%) than among BSI isolates (7.1%) (*p* = 0.0001). The *tet* genes were found in only two MRSA isolates. The *erm*T/*erm*C profile was associated with nasal isolates (91.3%) (*p* = 0.0001), while the *erm*T profile was the most frequent among BSI isolates (85.7%). All 106 (89.8%) isolates with the iMLS_B_ phenotype carried the *erm*T gene.

### Virulence Genes, IEC types and Clades

**Fig. 1:**
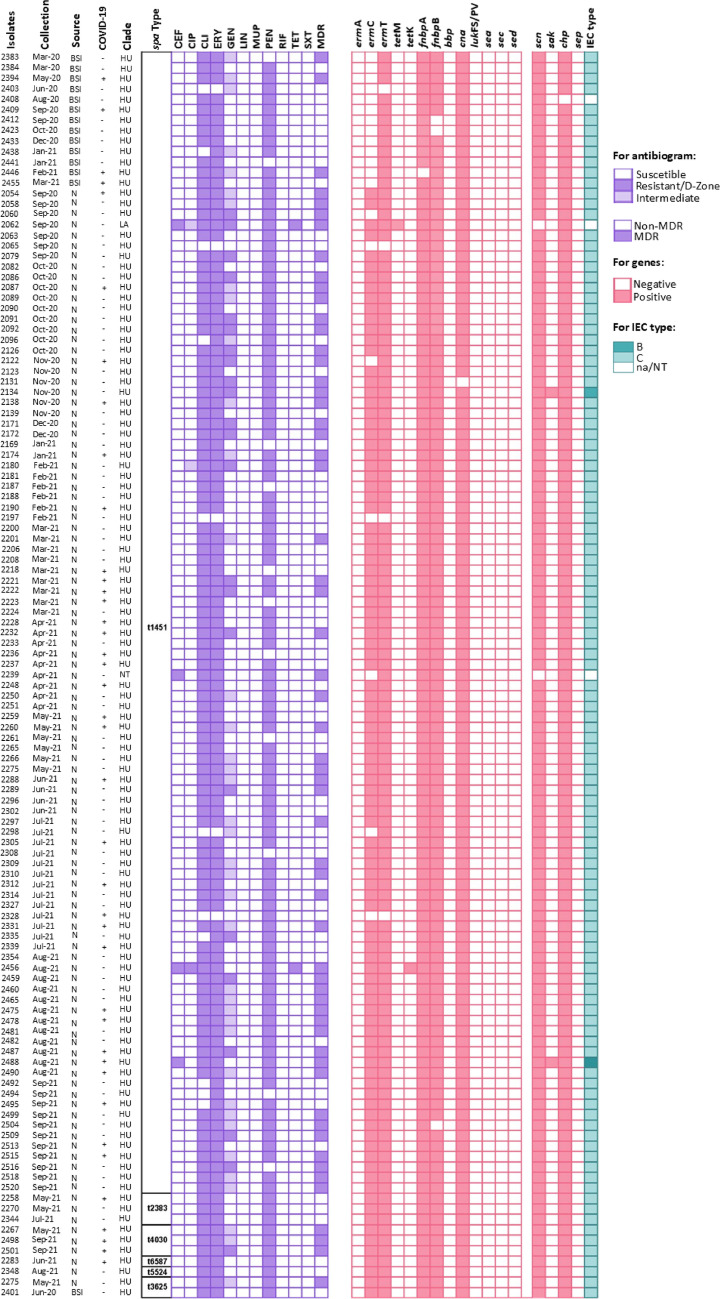
Heat map of clinical and microbiological profiles associated with 118 CC398 *Staphylococcus aureus* isolates from bloodstream infection and nasal swabs of hospitalized patients during the COVID-19 pandemic. CEF: Cefoxitin; CIP: Ciprofloxacin; CLI: Clindamycin; ERY: Erythromycin; GEN: Gentamicin; LIN: Linezolid. MUP: Mupirocin; PEN: Penicillin G; RIF: Rifampicin; TET: Tetracycline; SXT: Trimethoprim-sulfamethoxazole; na/NT: not applicable/not typable

The adhesion genes *fnb*A, *fnb*B and *cna* were detected in more than 95% of the isolates. The *bbp*, *sea*, *sec*, *sed*,* and luk*FS-PV were not found among the isolates (Fig. 1).

In this study, the IEC system (indicative of the presence of the prophage ΦSa3), which is characteristic of the HU clade, was detected in almost all isolates (98.3%, 116/118). The IEC type C, which presents the *scn* and *chp* genes, was the most frequently found (97.4%, 113/116) (Fig. 1; Table 3).

**Table 3:** Clades and their markers detected in 118 CC398 *Staphylococcus aureus* isolates from bloodstream infection and nasal swabs

	Clade (*N*/%)	Clinical source (*N*/%)	Clade marker genes		Resistance genes (%)	Virulence genes (%)	IEC marker genes				IEC type (%)	*spa* type (%)
			*tet*M	*scn*			*scn*	*chp*	*sak*	*sea/sep*		
MRSA (*n* = 4)	LA (1/25)	Nasal	+	-	*erm*C, *erm*T	*fnb*A, *fnb*B, *cna*	-	na	na	na	na	t1451
	HU (2/50)	Nasal	-	+	*erm*C, *erm*T	*fnb*A, *fnb*B, c*na*	+	+	+	-	B (50)	t1451
							+	+	-	-	C (50)	
	NT (1/25)	Nasal	-	-	*erm*T	*fnb*A, *fnb*B, *cna*	-	na	na	na	na	t1451
MSSA (*n* = 114)	HU (109/95.6)	BSI (12/85.7) Nasal (97/85)	-	+	*erm*C (87.1), *erm*T (100)	*fnb*A (97.2) *fnb*B (97.2) *cna* (99.1)	+	+	-	-	C (98.2)	t1451 (91.8) t4030 (2.7) t2383 (2.7) t3625 (1.8) t5524 (0.9) t6587 (0.9)
							+	+	+	-	B (0.9)	
							+	-	-	-	NT (0.9)	
	HU (4/3.5)	BSI (1/7.1) Nasal (3/2.6)	-	+	-	*fnb*A (100) *fnb*B (100) *cna* (100)	+	+	-	-	C (100)	t1451 (100)
	HU (1/0.9)	BSI (1/7.1)	-	+	*erm*T	*fnb*A, *fnb*B, *cna*	+	+	-	-	C	t1451

MRSA: Methicillin resistant *Staphylococcus aureus*; MSSA: Methicillin susceptible *Staphylococcus aureus*; LA: Livestock associated; HU: Human adapted; HU/LA: human adapted and livestock-associated; NT: non-typeable; BSI: Bloodstream infection; IEC: Immune evasion cluster; na: not applicable because isolates negative for the *scn* gene

Among the MRSA isolates, one carried the *scn*,* chp and sak* genes and was included in IEC type B, while another isolate presented the *scn* and *chp* genes, in addition to the *tet*K gene, and was included in IEC type C. A third MRSA isolate was included in the LA clade (absence of IEC and presence of *tet*M gene). The clades presented the adhesins *fnb*A, *fnb*B, and *cna* in most isolates.

### STs and *spa* Types

All nine *S. aureus* isolates selected for MLST presented ST398, four of the *spa* type t1451, and five of each of the other *spa* types found in the study. The *spa* type t1451 was prevalent in both BSI and nasal colonization (91.5%, 108/118). The least frequent types were: t2383 (*n* = 3; 2.5%), t4030 (*n* = 3; 2.5%), t3625 (*n* = 2; 1.7%), t5524 (*n* = 1; 0.8%) and t6587 (*n* = 1; 0.8%), and were primarily found among nasal isolates (Fig. 1). The distribution of *spa* types during the study months shows that, except for one isolate, *sp*a type t1451 was the only detected among BSI isolates. Although this spa type was also prevalent among nasal colonization isolates, starting in May/2021 there was an increase in the diversity of spa types among these isolates (Supplementary Fig. 1).

Among the 116 isolates of the prevalent HU clade, the profile HU-MSSA/t1451/*erm*C+/*erm*T+/PVL-/IEC type C was found in 87 (75%) isolates, 85 from nasal swab and two isolates from BSI. The LA-MRSA isolate presented the profile t1451/*erm*C+/*erm*T+/*tet*M+/PVL-.

## Discussion

Although CC398 is the predominant MRSA lineage associated with livestock [4, 6], MSSA isolates from this clonal complex have emerged as a growing cause of human infections [5, 10]. In this study, we characterized 118 CC398 *S. aureus* isolates collected in a Brazilian hospital during the COVID-19 pandemic, predominantly human associated and presenting multidrug resistance in almost 50% of isolates. This lineage was frequent among the patients, with rates of 40% among MSSA isolates in both bloodstream and nasal isolates, possibly reflecting the increase in antimicrobial use during the pandemic [16]. Another challenge was the initial difficulty in implementing more effective infection control and prevention measures [34]. In China, among 227 isolates recovered from clinical sources in two periods prior to the pandemic, 2013–2014 and 2018–2019, the prevalence of the CC398-MSSA lineage increased from 5.5% to 18.4% between the periods [11]. Bouiller et al. in France detected, between 2019 and 2020, 24.6% of individuals in the community and 18.3% of hospitalized patients as carriers of this pathogen [12]. Although Di Gregorio et al. [10] recently identified CC398 as the predominant MSSA lineage in BSI in a multicenter study in South American countries, reports on CC398 *S. aureus* in our country are still rare and had not yet been described during the COVID-19 pandemic. In addition, these studies are focused on colonization and have shown low frequency of CC398, such as 0.3% among children [14], and 4.1% and 6.6% among MRSA and MSSA isolates, respectively, in diabetic adults [35]. Therefore, our results suggest that the high frequency of CC398 isolates found may be related to the impact caused by the COVID-19 pandemic, due to the selective pressure resulting from the increased use of antimicrobials, as well as the difficulties in maintaining effective prevention and control measures during this period.

Resistance rates above 90% to erythromycin and clindamycin, as well as the iMLS_B_ phenotype, detected in approximately 90% of the isolates may be related to the widespread use of antimicrobials, particularly those from the MLSB group (macrolides, lincosamides, and streptogramin B), during the pandemic. Macrolides were the most prescribed antibiotics for COVID-19 patients in North America and ranked second in Europe [15]. In Brazil, data show a sharp increase in azithromycin consumption between January and April 2020, placing it as the second most frequently prescribed class of antibiotic in Rio de Janeiro [16]. A comparable trend was observed at the community level, with a significant increase in azithromycin sales in Brazilian pharmacies throughout the pandemic period [36]. It is also important to emphasize that our study showed that the prior use of antimicrobials, including azithromycin, was more frequent among patients with COVID-19. Indeed, in the present study, *ermT* and *ermC* genes were frequently detected among the isolates, with rates of 96.6% and 83.1%, respectively, in addition to iMLS_B_ phenotype, which was detected in almost 90% of the isolates. In a study involving invasive infections, Mama et al. [5] proposed that the iMLSB phenotype and the presence of the *ermT* gene could serve as markers of MSSA-CC398 strains. It is worth noting that CC398 isolates showed significantly greater resistance to clindamycin and the presence of the iMLS_B_ profile compared to non-CC398 isolates, when evaluated in a larger project developed by our group (data not shown). The results support, in part, the hypothesis that the extensive use of macrolides during the pandemic on MSSA-CC398 isolates that already exhibited resistance to these antimicrobials may have contributed to the spread of the lineage within our hospital.

The high frequency of MSSA-CC398 observed in this study may also be associated with the presence of genetic determinants that increase its adaptation to the human host. Uhlemann et al. [37] showed that human isolates of CC398-MSSA exhibited greater adhesion to human keratinocytes and keratin compared to porcine isolates, suggesting specific characteristics that favor nasal and cutaneous colonization in humans. Another factor that potentially contributes to this adaptive advantage is the acquisition of the ϕSa3 prophage, which carries the immune evasion cluster (IEC). Kashif et al. [38] demonstrated that CC398 carrying the ϕSa3 prophage are more virulent and better equipped for specific adaptation to humans. In our study, the IEC was detected in most isolates (98.3%), indicating the possibility of human-to-human transmission within the hospital.

In our study, *spa* type t1451 was the most frequently identified among both MSSA and MRSA isolates. This *spa* type has been predominantly associated with CC398-MSSA in South America, including Brazil and Paraguay, and is also commonly found in Argentina [16], being rarely detected in MRSA-CC398 isolates [5, 10]. Throughout the study period, *spa* type distribution remained stable among the isolates, with t1451 consistently prevailing. In early May, an increase in *spa*-type diversity was observed among nasal isolates, possibly related to the increase in hospital admissions during this period. It is worth noting that CC398 isolates (*spa* type t1451), originating from BSI and nasal colonization from the same patient, were obtained in October 2020, and the patient remained colonized until February 2021 (data not shown), highlighting the clinical relevance of this lineage and its persistence in the hospital environment.

Genotypic analysis confirmed that the most prevalent profile was MSSA/t1451/*erm*C+/*erm*T+/PVL-/IEC type C (75%), classifying the isolates within the HU clade, characterized by the presence of IEC and the absence of *tet*M gene. These findings are in line with the results of Di Gregorio et al. [10], who analyzed 404 CC398-MSSA genomes from a multicenter study on *S. aureus* BSI from 58 hospitals across South America. The recurrence of this profile corroborates the hypothesis that this lineage, already circulating in Brazil before the pandemic, may have been better selected under the selective pressure imposed by the increased use of macrolides. Given its evolutionary dynamics, it is plausible that this lineage has increased its ability to persist and spread in both hospital and community settings, considering its attributes that facilitate adaptation to the human host.

In this study, only one isolate with LA-MRSA profile (livestock associated) was identified. The colonized patient resides in an urban area, and no information about their occupation was available. However, LA-MRSA strains can be indirectly transmitted in the community through social contact, contaminated surfaces, or individuals exposed to animals, which can act as vectors of CC398 in other environments, such as urban areas [9].

Limitations of this study include the fact that we evaluated CC398 isolates from only one hospital. Other factors that could influence the acquisition of this microorganism, such as underlying diseases, previous hospitalizations, and comorbidities were not evaluated.

## Conclusions

*S. aureus* CC398 lineage isolates from the human clade, carrying the IEC type C and exhibiting multidrug resistance, were frequent among colonized patients and those with bloodstream infection during the COVID-19 pandemic. The antimicrobial selective pressure exerted on these isolates, previously resistant to macrolides, may have favored their dissemination in the environment, highlighting the importance of effective prevention and control measures, particularly during pandemic periods.

## Supplementary Information

Below is the link to the electronic supplementary material.

Supplementary Material 1

## Data Availability

The data sets generated and analyzed during the current study, such as the PFGE and MLST are not publicly available as there is no public database to deposit PFGE results and no new ST was found in the present study. However, these data are available from the corresponding author on request.
