# Supplementary material for: Staphylococcus aureus CC398 Lineage of the Human Clade Isolated from Bloodstream Infection and Colonization and Spread among Brazilian Patients Hospitalized during the COVID-19 Pandemic
Source: Curr Microbiol. 2026 Jun 30;83(8):459. doi: 10.1007/s00284-026-04996-x (PMC13319229; doi:10.1007/s00284-026-04996-x)
Supplement: Supplementary file 1 — Supplementary Material 1 [file 284_2026_4996_MOESM1_ESM.docx]

**Supplementary Materials**


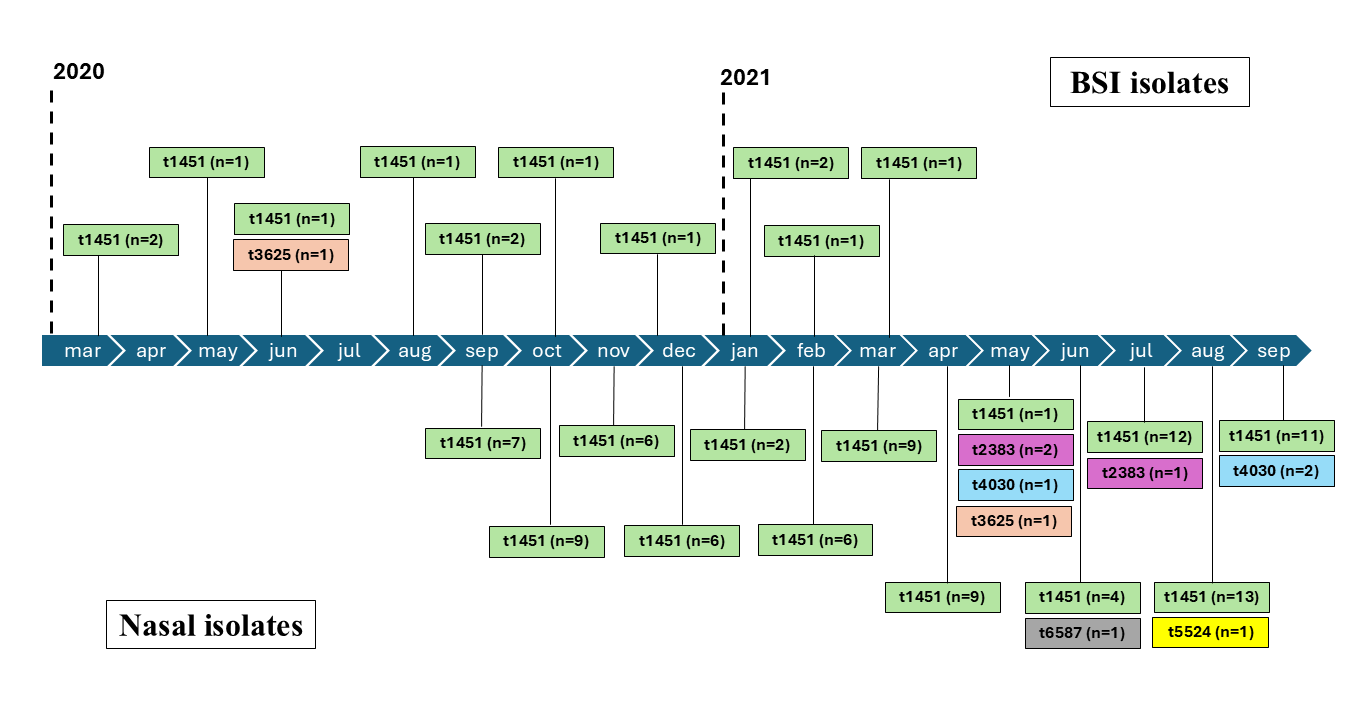


**Supplementary Figure 1.** Timeline of the distribution of *spa types* (number of isolates) in 118 CC398 *Staphylococcus aureus* isolates from bloodstream infection and nasal swabs between March 2020 and September 2021. BSI: Bloodstream infection.

**Supplementary table 1.** Resistance and virulence genes, oligonucleotides and PCR conditions used in this study to investigate 18 genes in CC398 *Staphylococcus aureus*.

| **Gene** | **Oligonucleotides- Sequence (5'-3')** | **Concentration**  **(uM)** | **Cycle^a^** | **Amplicon**  **(bp)** | **Reference** |
| --- | --- | --- | --- | --- | --- |
| *erm*A | GTTCAAGAACAATCAATACAGAG | 0.8 | 1 | 421 | [23] |
|  | GGATCAGGAAAAGGACATTTTAC |  |  |  |  |
| *erm*B | GCATTTAACGACGAAACTGGCT | 0.8 | 2 | 573 | [24] |
|  | GACAATACTTGCTCATAAGTAATGGT |  |  |  |  |
| *erm*C | GCTAATATTGTTTAAATCGTCAATTCC | 0.8 | 3 | 572 | [23] |
|  | GGATCAGGAAAAGGACATTTTAC |  |  |  |  |
| *erm*T | CCGCCATTGAAATAGATCCT | 0.8 | 4 | 200 | [8] |
|  | TTCTGTAGCTGTGCTTTCAAAAA |  |  |  |  |
| *tet*M | AGTGGAGCAATTACAGAA  CATATGTCCTGGCGTGTCTA | 0.8 | 5 | 169 | [25] |
| *tet*K | GTAGCGACAATAGGTAATAGT  GTAGTGACAATAAACCTCCTA | 0.8 | 6 | 360 | [26] |
| *fnbp*A | CACAACCAGCAAATATAG  CTGTGTGGTAATCAATGTC | 1.6 | 7 | 1362 | [27] |
| *fnbp*B | GTAACAGCTAATGGTCGAATTGATACT | 1.6 | 8 | 524 | [28] |
|  | CAAGTTCGATAGGAGTACTATGTTC |  |  |  |  |
| *bbp* | AACTACATCTAGTACTCAACAACAG | 1.6 | 8 | 575 | [28] |
|  | ATGTGCTTGAATAACACCATCATCT |  |  |  |  |
| *cna* | GTCAAGCAGTTATTAACACCAGAC | 1.6 | 8 | 423 | [28] |
|  | AATCAGTAATTGCACTTTGTCCACTG |  |  |  |  |
| *luk*FS-PV | ATCATTAGGTAAAATGTCTGGACATGATCCA | 1 | 8 | 433 | [29] |
|  | GCATCAASTGTATTGGATAGCAAAAGC |  |  |  |  |
| *scn* | AGCACAAGCTTGCCAACATCG  TTAATATTTACTTTTTAGTGC | 0.8 | 9 | 257 | [8] |
| *sea* | AGATCATTCGTGGTATAACG | 0.8 | 9 | 408 | [8] |
|  | TTAACCGAAGGTTCTGTAGA |  |  |  |  |
| *sec* | CTTGTATGTATGGAGGAATAACAA | 1 | 10 | 283 | [30] |
|  | TGCAGGCATCATATCATACCA |  |  |  |  |
| *sed* | GTGGTGAAATAGATAGGACTGC | 1 | 11 | 384 | [30] |
|  | ATATGAAGGTGCTCTGTGG |  |  |  |  |
| *sep* | AATCATAACCAACCGAATCA  TCATAATGGAAGTGCTATAA | 0.8 | 12 | 500 | [8] |
| *chp* | GAAAAAGAAATTAGCAACAACAG  CATAAGATGATTTAGACTCTCC | 0.8 | 13 | 405 | [31] |
| *sak* | AAGGCGATGACGCGAGTTAT  GCGCTTGGATCTAATTCAAC | 0.8 | 14 | 203 | [8] |

**Cycles:** **1**: 95°C/30s; 54°C/30s; 72°C/1min - 30x; **2**: 95°C/30s; 58°C/45s; 72°C/1min - 30x; **3**: 94°C/30s; 57°C/30s; 72°C/1min - 30x; **4:** 94°C/30s; 53°C/30s; 72°C/30s - 30x; **5:** 94°C/30s; 58°C/30s; 72°C/30s - 30x; **6:** 94°C/30s; 55°C/30s; 72°C/45s - 30x; **7:** 94°C/1min; 50°C/1min 72°C/2min - 30x; **8:** 94°C/1min; 55°C/1min; 72°C/1min - 30x; **9:** 94°C/30s; 50°C/30s; 72°C/1min - 30x; **10:** 94°C/1min; 63°C/45s; 72°C/1min - 30x; **11:** 94°C/1min; 64°C/1min; 72°C/1min - 30x; **12:** 94°C/30s; 50°C/45s; 72°C/45s - 30x; **13:** 94°C/30s; 51°C/30s; 72°C/45s - 30x; **14:** 94°C/30s; 52°C/30s; 72°C/30s - 30x.

**Supplementary table 2.** Antimicrobial resistance in 118 CC398 *Staphylococcus aureus* isolates from bloodstream infection and nasal swabs according to the diagnosis of COVID-19

|  | **N (%) of isolates** | | | | | | |
| --- | --- | --- | --- | --- | --- | --- | --- |
| **Resistance characteristics** |  | **BSI** | | | **Nasal swab** | | |
|  | **Total**  **(n=118)** | **Total**  **(n=14)** | **COVID-19** | | **Total**  **(n=104)** | **COVID-19** | |
|  |  |  | **Positive**  **(n=4)** | **Negative**  **(n=10)** |  | **Positive**  **(n=36)** | **Negative**  **(n=68)** |
| **Resistance^a^** |  |  |  |  |  |  |  |
| Cefoxitin | 4 (3.4) | - | - | - | 4 (3.9) | 1 (2.7) | 3 (4.4) |
| Ciprofloxacin | 3 (2.6) | - | - | - | 3 (2.9) | - | 3 (4.4) |
| Clindamycin | 107 (90.7) | 12 (85.7) | 4 (100) | 8 (80) | 95 (91.3) | 35 (97.2) | 60 (88.2) |
| iMLS_b_ phenotype | 106 (89.8) | 12 (85.7) | 4 (100) | 8 (80) | 94 (90.4) | 34 (94.4) | 60 (88.2) |
| Erythromycin | 113 (95.8) | 13 (92.8) | 4 (100) | 9 (90) | 100 (96.1) | 35 (97.2) | 65 (95.6) |
| Gentamycin | 61 (51.7) | 5 (35.7) | 2 (50) | 3 (30) | 56 (53.8) | 20 (55.5) | 36 (53) |
| Penicillin | 109 (92.4) | 13 (92.8) | 4 (100) | 9 (90) | 95 (91.3) | 33 (91.7) | 62 (91.2) |
| Tetracycline | 3 (2.6) | - | - | - | 3 (2.9) | 1 (2.8) | 2 (2.9) |
| **MDR isolates** | 58 (49.2) | 3 (21.4) | 2 (100) | 1 (10) | 55 (52.9) | 21 (58.3) | 34 (50) |

^a^ isolates classified as resistant and intermediate resistant, according to CLSI, 2020; BSI: Bloodstream infection; iMLS_b_: inducible macrolide-lincosamide-streptogramin B resistance.
